# Prevalence, mortality and risk factors for self-reported COPD among smokers and never smokers, NHANES 1999-2018

**DOI:** 10.18332/tid/192745

**Published:** 2024-09-18

**Authors:** Xiaohua Li, Minwei Xue, Donggang Xu, Caiyun Fan, Jianquan Zhang

**Affiliations:** 1 Department of Respiratory and Critical Medicine, the Eighth Affiliated Hospital, Sun Yat-Sen University, Shenzhen, China; 2 School of Statistics and Information, Shanghai University of International Business and Economics, Shanghai, China; 3 Second Clinical Department, Shengjing Hospital, China Medical University, Shenyang, China

**Keywords:** chronic obstructive pulmonary disease, smokers, prevalence, National Health and Nutrition Examination Survey, risk factors

## Abstract

**INTRODUCTION:**

Cigarette smoke is the main risk factor for chronic obstructive pulmonary disease (COPD), but 25% to 50% of cases occur in non-smokers. In the US, limited recent national data compare COPD prevalence between smokers and never smokers. Furthermore, our study seeks to explore the prevalence and mortality of self-reported COPD among smokers (including current smokers and ex-smokers) and never smokers in the US from 1999 to 2018, and to identify the risk factors and differences.

**METHODS:**

This cross-sectional analysis used data from the National Health and Nutrition Examination Survey (NHANES) 1999–2018. Age-standardized prevalence of self-reported COPD among current smokers, ex-smokers, and never smokers was calculated using sample weights and 2010 US Census estimates. Risk factors were evaluated through weighted logistic regression models. Subsequently, participants who enrolled in the study cohort were followed until 31 December 2019, to determine all-cause mortality rates.

**RESULTS:**

Between 1999 and 2018, the weighted prevalence of COPD among current smokers, ex-smokers, and never smokers in the U.S. was 12.6%, 9.6%, and 4.1%, respectively. The mortality rates observed were 21.1% among current smokers with COPD, 29% among ex-smokers with COPD, and 12% among never smokers with COPD. Over this period, among the general population in the U.S., the proportion of current smokers has declined, the proportion of never smokers has increased, and the proportion of ex-smokers has remained relatively stable. From 1999 to 2018, COPD prevalence rose from 13.7% to 21.9% among current smokers, stayed at 10.1% among ex-smokers, and dropped from 4.9% to 3.3% among never smokers. Independent risk factors for COPD across all groups included being female, older, and lower income. In particular, US citizens and non-Hispanic Whites (among ex-smokers and never smokers) were at higher risk compared to their counterparts.

**CONCLUSIONS:**

The prevalence and all-cause mortality of COPD among current smokers and ex-smokers remain elevated. Although the prevalence of COPD among never smokers is gradually declining, it continues to be significant, thereby maintaining a substantial burden of disease. Furthermore, common independent risk factors for COPD across current smokers, ex-smokers, and never smokers include female gender, advanced age, lower income, and deviations from normal body weight whether overweight or underweight.

## INTRODUCTION

Chronic obstructive pulmonary disease (COPD) is characterized as a chronic respiratory disease with progressive and irreversible airflow limitations. The global prevalence of COPD stands at 13.1%, with a rising trend^1^. COPD is also the third-leading cause of death globally^2^ and is projected to increase in the next 40 years, leading to approximately 5 million deaths each year^3^. Although smoking is the primary risk factor for COPD, it is not the sole determinant. In 2019, non-smokers may constitute about one-third of the 391.9 million people worldwide aged 30–79 years with COPD^4,5^.

In addition to cigarette smoking, several studies have highlighted biomass fuels, air pollutants, workplace dust and fumes, a history of respiratory infections, poor nutrition, and socioeconomic disadvantage as notable risk factors for COPD^6,7^. Consequently, there is a growing importance in investigating COPD among smokers and never smokers within the field of global public health. However, there persists a dearth of knowledge regarding never-smoking COPD, with only a limited number of studies conducted on the prevalence and mortality of COPD among smokers and never smokers^8-10^. Therefore, it is essential to undertake additional research on the epidemiology of COPD among smokers and never smokers, to understand disease trends better and inform strategies for prevention and treatment.

The National Health and Nutrition Examination Survey (NHANES) is distinguished by its substantial and nationally representative sample size. This study aimed to examine the prevalence trend of self-reported COPD among smokers (including current smokers and ex-smokers) and never smokers aged 20–79 years in the US, along with its associated risk factors, using data from the NHANES collected between 1999 and 2018. Furthermore, a cohort study was carried out to investigate the all-cause mortality from self-reported COPD among current smokers, ex-smokers, and never smokers by linking NHANES data with mortality data from the National Death Index (NDI) in 2019.

## METHODS

### Data and study population

NHANES, conducted by the National Center for Health Statistics (NCHS), employs a nationally representative cross-sectional design utilizing a complex, multi-stage, stratified, clustered probability sampling method. Approval for the study was obtained from the NCHS Ethics Review Board (ERB), and all participants provided informed consent^11^. The data, publicly accessible on the Centers for Disease Control and Prevention (CDC) website^12^, encompassed health interviews conducted in respondents’ residences and health measurements obtained at mobile examination centers (MECs). Our study utilized data from 10 successive survey cycles of the NHANES (1999–2018, two-year cycle) and adhered to the Strengthening the Reporting of Observational Studies in Epidemiology (STROBE) guidelines.

### Definition of main variables

Using data from the National Health and Nutrition Examination Survey (NHANES), we included adults aged 20–79 years in our study. Never smokers were identified based on their responses to the question: ‘Have you smoked at least 100 cigarettes in your entire life?’. Participants who answered affirmatively were classified as smokers, while those who answered negatively were classified as never smokers. Subsequently, we further assessed smoking status with the question: ‘Do you now smoke cigarettes?’. Smokers who responded ‘every day’ or ‘some days’ were categorized as current smokers, whereas those who responded ‘not at all’ were classified as ex-smokers. The definition of COPD in this study was based on the following question: ‘Has a doctor or other health professional ever told you that you had emphysema/chronic bronchitis/COPD?’. A positive response to any part of this question was necessary to classify a case of self-reported COPD.

### Other covariates

In addition to collecting data on smoking status and COPD, demographic characteristics were extracted, encompassing variables such as sex, age (20–39, 40–59, 60–79 years), race/ethnicity (non-Hispanic White, non-Hispanic Black, Hispanic, Mexican American, other), citizenship status (US citizen, non-US citizen), an education level (lower than high school, high school or higher), body mass index (BMI, kg/ m^2^) categories (underweight: <18.5, normal weight: 18.5–24.9, overweight: 25.0–29.9, obese: ≥30.0), and poverty-to-income ratio (PIR) classifications (low level: PIR <2, medium level: PIR=2–4, high level: PIR >4).

Demographic information, including sex, age, race/ethnicity, citizenship status, and education level, was self-reported by participants in the questionnaire section. BMI was determined by trained health professionals at the Mobile Examination Center (MEC) using a standardized protocol, calculated as an individual’s weight (kg) divided by the square of their height (m).

The PIR was computed as the total household income divided by the poverty threshold defined by the US Census Bureau, which is an index developed by NHANES to reflect the annual income of a household adjusted for family size and established poverty threshold guidelines set by the US Department of Health and Human Services (HHS)^13^. A higher PIR indicates a higher income level.

### Mortality follow-up

The NHANES data were cross-referenced with death records in the National Death Index (NDI) from 1999 through 31 December 2019 using a rigorous probabilistic matching process and review of death certificates based on seven matching criteria, such as Social Security number, sex, and date of birth. The follow-up period for each study participant was defined as the duration between the NHANES baseline examination and the date of the participant’s death or the last follow-up visit (31 December 2019). The primary outcome measure in this study was allcause mortality, which was defined as death resulting from any cause. For further details on pertinent mortality records and definitions of causes of death, please refer to the following resources: https://www.cdc.gov/nchs/data-linkage/mortality-public.htm.

### Statistical analysis

We performed a descriptive analysis of demographic characteristics using R software (V.4.2.2). The Rao-Scott chi-squared test was utilized to assess the significance of the association between each demographic variable and COPD, and the weights provided by NHANES were integrated into the statistical analysis of the NHANES sample to adjust for the complex survey design. Our study also examined COPD prevalence in various demographic groups from 1999 to 2018 and standardized COPD prevalence using age data from the 2010 US Census, which involved stratifying into three age groups (20–39, 40–59, and 60–79 years) and calculating age-standardized prevalence for each survey period. Lastly, proportional estimations of demographic variables were calculated along with their corresponding 95% confidence intervals.

To explore potential explanatory factors such as demographic variables, we employed weighted univariate logistic regression models to analyze and identify variables with significant impacts. Four nested logistic regression models were constructed in these groups, each building upon the previous one by adding sequentially explanatory variables: sex, age, and race/ethnicity in Model 1, plus citizenship in Model 2, plus BMI and PIR in Model 3, plus education level in Model 4. Parameter estimation, significance tests for regression parameters, multicollinearity test, and the likelihood ratio test were performed.

Parameters with two-tailed p<0.05 were considered statistically significant. Finally, Model 3 was chosen for the current and never smokers, while Model 4 was chosen for the ex-smokers, and odds ratios with corresponding 95% confidence intervals were calculated for each parameter (Supplementary file).

Participants’ survival time was calculated from their baseline medical examination until death, loss to follow-up, or 31 December 2019. In addition to calculating the survival rates, the study also determined medians and quartiles of survival time. A Kaplan-Meier plot was created for current smokers, ex-smokers, and never smokers to examine the impact of COPD on all-cause mortality. Then, the log-rank test was used to assess the statistical significance of differences in survival rates between COPD and nonCOPD groups (Supplementary file).

## RESULTS

### Characteristics of study participants

**Figure 1:**
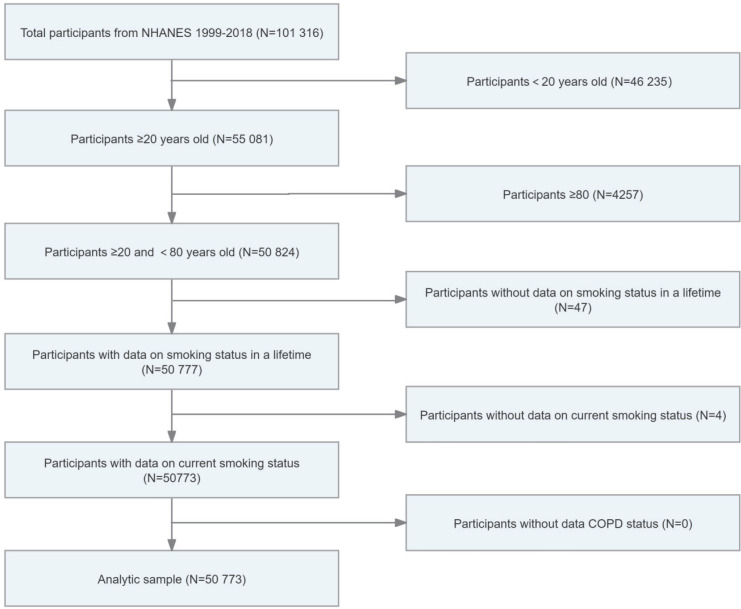
Flow chart of current smokers, ex-smokers, and never smokers, aged 20–79 years, NHANES 1999–2018, United States (N=50773)

The study included a sample of 50773 individuals aged 20–79 years (Figure 1). Of these participants, 11257 were categorized as current smokers (weighted n = 46600007), 11865 were categorized as ex-smokers (weighted n = 49512069), and 27651 were categorized as never smokers (weighted n = 11378367). The participants, categorized as current smokers, ex-smokers, and never smokers, were subsequently divided into two subgroups (COPD and non-COPD). Current smoking-related COPD was identified in 12.59% (n=1374) of current smokers, while ex-smoking-related COPD was observed in 9.61% (n=1210) of ex-smokers. Additionally, never smoking-related COPD accounted for 4.14% (n=1109) of never smokers. The proportions of current smoking COPD (n=789; 62.2%), ex-smoking-related COPD (n=576; 51.1%), and never smoking-related COPD (n=829; 75%) were higher in female patients compared to male patients. Among never smokers, female COPD patients (n=829; 75%) outnumbered male patients (n=280; 25%) by three times.

It is noteworthy that the age distribution of COPD prevalence varies by smoking status. Specifically, most individuals with current smoking-related COPD were aged 40–59 years (n=619; 48.9%), whereas the majority of those with ex-smoking-related COPD were aged 60–79 years (n=804; 56.7%). Furthermore, non-Hispanic Whites exhibited a higher proportion of COPD among current smokers (n=866; 78.9%), ex-smokers (n=752; 81.4%), and never smokers (n=510; 70.3%). However, no significant difference was observed among never smokers (p=0.677). Among current smokers, ex-smokers, and never smokers, demographic disparities in citizenship status, BMI, and PIR were observed between COPD and non-COPD subgroups. Notably, 98.2% of current smokers with COPD were US citizens (n=1330). COPD with low PIR (<2) was higher in current smokers (n=869; 58.3%), ex-smokers (n=629; 56.3%), and never smokers (n=531; 40.3%). Obese COPD (BMI ≥30) accounted for more than 50% of ex-smokers (n=583; 51.5%) and never smokers (n=561; 53.7%) (Table 1).

**Table 1:** Demographic characteristics of self-reported COPD and non-COPD among current smokers, ex-smokers, and never smokers, aged 20–79 years, NHANES 1999–2018, United States (N=50773)

*Characteristics*	*Current smokers n (weighted %)*			*Ex-smokers n (weighted %)*			*Never smokers n (weighted %)*		
	*COPD (N=1374)*	*Non-COPD (N=9883)*	*p*	*COPD (N=1210)*	*Non-COPD (N=10655)*	*p*	*COPD (N=1109)*	*Non-COPD (N=26542)*	*p*
**Sex**			<0.001			<0.001			<0.001
Male	585 (37.8)	5878 (57.0)		634 (48.9)	6470 (57.8)		280 (25.0)	10660 (43.0)	
Female	789 (62.2)	4005 (43.0)		576 (51.1)	4185 (42.2)		829 (75.0)	15882 (57.0)	
**Age** (years)			<0.001			<0.001			<0.001
20–39	322 (26.0)	4414 (49.0)		112 (12.0)	2309 (24.9)		285 (26.8)	11371 (44.0)	
40–59	619 (48.9)	3670 (40.0)		294 (31.3)	3493 (41.4)		377 (39.8)	8699 (37.8)	
60–79	433 (25.1)	1799 (11.0)		804 (56.7)	4853 (33.7)		447 (33.4)	6472 (18.1)	
**Race**			<0.001			<0.001			<0.001
Mexican American	79 (2.1)	1489 (7.4)		105 (3.2)	1942 (7.1)		149 (4.4)	5543 (10.1)	
Other Hispanic	72 (3.8)	668 (5.1)		71 (2.7)	878 (4.8)		107 (5.4)	2550 (6.7)	
Non-Hispanic White	866 (78.9)	4496 (67.7)		752 (81.4)	5295 (76.1)		510 (70.3)	9211 (61.8)	
Non-Hispanic Black	267 (9.4)	2543 (13.6)		214 (7.3)	1792 (7.1)		256 (12.4)	5968 (12.8)	
Other	90 (5.7)	687 (6.2)		68 (5.4)	748 (5.0)		87 (7.3)	3270 (8.6)	
**Education level**			0.031			<0.001			0.68
Lower than high school	479 (28.3)	3298 (25.1)		374 (31.0)	2808 (15.2)		261 (14.4)	6354 (13.9)	
High school or higher	893 (71.7)	6572 (74.9)		833 (69.0)	7842 (84.8)		846 (85.6)	20150 (86.1)	
**PIR**			<0.001			<0.001			<0.001
<2	869 (58.3)	5340 (46.7)		629 (56.3)	3973 (26.6)		531 (40.3)	10330 (30.9)	
2–4	257 (26.9)	2192 (29.3)		291 (26.0)	2713 (30.5)		251 (27.8)	6515 (28.0)	
>4	124 (14.8)	1456 (24.0)		198 (17.7)	3062 (43.0)		235 (32.0)	7077 (41.1)	
**Citizenship status**			<0.001			<0.001			<0.001
US citizen	1330 (98.2)	8713 (92.2)		1156 (95.9)	9360 (92.9)		1010 (95.7)	21356 (88.0)	
Non-US citizen	44 (1.8)	1145 (7.8)		50 (4.1)	1281 (7.1)		98 (4.3)	5105 (12.0)	
**BMI**			<0.001			<0.001			<0.001
<18.5	70 (5.5)	253 (2.5)		12 (1.1)	80 (0.9)		13 (1.1)	334 (1.5)	
18.5–24.9	386 (30.4)	3200 (35.9)		191 (16.9)	2223 (23.9)		186 (17.9)	7033 (30.0)	
25.0–29.9	340 (25.9)	2985 (31.8)		345 (30.5)	3730 (36.3)		283 (27.3)	8287 (32.8)	
≥30	500 (38.2)	2854 (29.7)		583 (51.5)	4022 (39.0)		561 (53.7)	9393 (35.7)	

P-value calculated using the chi-squared test. PIR: poverty-to-income ratio. BMI: body mass index (kg/m^2^).

### The distribution of current smokers, ex-smokers, and never smokers within both the general population and the population with COPD

**Figure 2:**
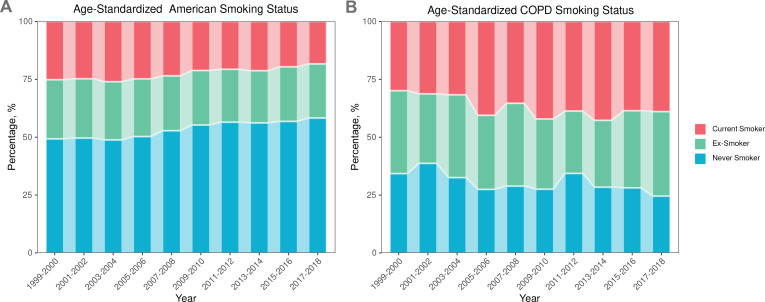
Age-standardized percentage of current smokers, ex-smokers, and never smokers among general population and the population with COPD, NHANES 1999–2018, United States

Between 1999 and 2018, the distribution of smoking status within the overall population underwent significant changes. Specifically, the proportion of current smokers declined, whereas the proportion of never smokers had an upward trajectory (Figure 2A). In the population with COPD, the proportion of current smokers and ex-smokers is notably high. Specifically, in 2018, the percentage of current smokers among COPD patients reached 38.9%. Conversely, the proportion of never smokers within this population exhibited a downward trend between 1999 and 2018 (Figure 2B).

### Prevalence of COPD among current smokers, ex-smokers, and never smokers

**Figure 3:**
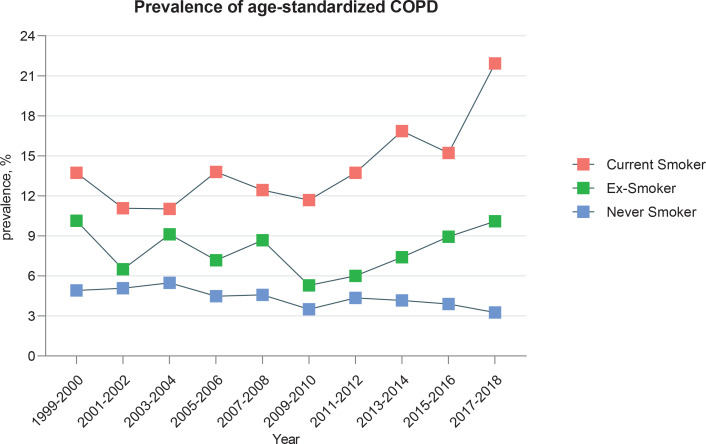
Age-standardized prevalence of self-reported COPD among current-smokers, ex-smokers, and never smokers, aged 20–79 years, NHANES 1999–2018, United States (N=50773)

Between 1999 and 2018, the crude and age-standardized prevalence rates of COPD among current smokers were 12.2% (n=1374) and 12.6% (n=1374), respectively. Among ex-smokers, these rates were 10.2% (n=1210) and 9.6% (n=1210), respectively. In never smokers, the crude and age-standardized prevalence rates of COPD were 4.0% (n=1109) and 4.1% (n=1109), respectively. The prevalence of COPD among current smokers exhibited a higher overall rate compared to ex-smokers and never smokers, with an increasing trend from 13.7% (n=96) in 1999 to 21.9% (n=192) in 2018. The prevalence of COPD among ex-smokers demonstrated a fluctuating upward trend, remaining relatively stable at 10.1% in both 1999 (n=115) and 2018 (n=180). Conversely, the prevalence of COPD among never smokers showed an overall decline, decreasing from 4.9% (n=321) in 1999 to 3.3% (n=493) in 2018 (Figure 3). For current smokers, disease incidence shows an overall increasing trend year by year by age, gender, race, education level, income level, citizenship status, and BMI. In contrast, disease rates among ex-smokers remained relatively stable. However, certain subgroups have notable fluctuations, particularly among older individuals (60–79 years), those with moderate income levels (PIR=2–4), and US citizens. These subgroups have shown an upward trend in disease incidence in recent years. In addition, among non-smokers, there was a significant decrease in the prevalence of COPD among women, US citizens, and individuals with low PIR levels (Supplementary file).

### All-cause mortality of COPD among current smokers, ex-smokers, and never smokers

**Figure 4:**
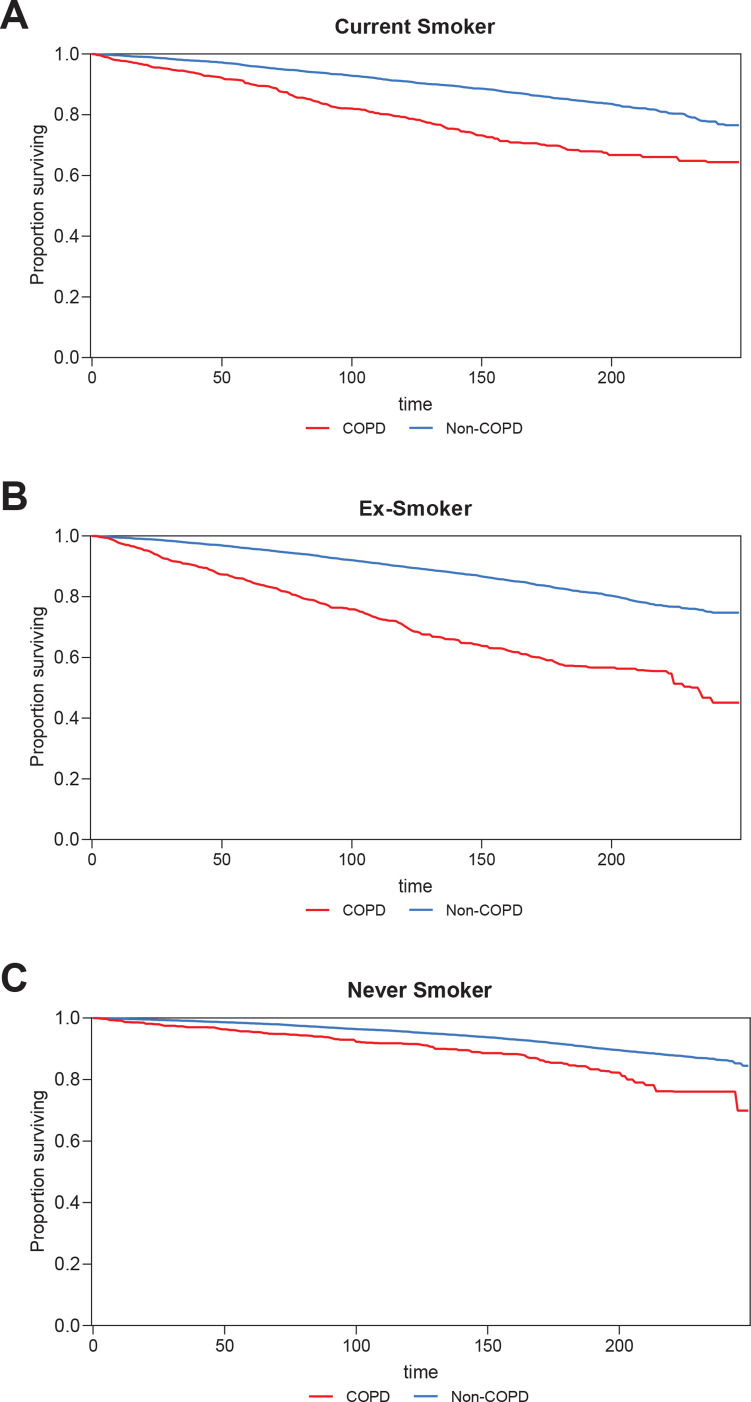
All-cause mortality between individuals with COPD and those without COPD among: A) Current Smoker, B) Ex-smoker, and C) Never Smoker, aged 20–79 years, NHANES 1999–2018, United States (N=50773)

The sample for the all-cause mortality analysis included 50773 individuals aged 20–79 years, comprising current-smokers (n=11257), ex-smokers (n=11865), and never smokers (n=27651), drawn from the NHANES (1999–2018). The follow-up period was extended until 31 December 2019. Among the cohort of current smokers, a total of 336 patients with COPD succumbed, accounting for 21.1%, while 1362 non-COPD patients, accounting for 11.2%, also died. In the group of ex-smokers, there were 430 deaths attributed to COPD, constituting 29% of the COPD cases, alongside 1851 deaths among individuals without COPD, which corresponds to 12.3%. Among never smokers, 156 COPD patients (12%) and 2069 individuals without COPD (5.7%) died. The median follow-up period for the study was 114 months (IQR: 62–171.25). Kaplan-Meier survival curves illustrate a statistically significant difference in all-cause mortality between individuals with COPD and those without COPD among current smokers (Figure 4A), ex-smokers (Figure 4B), and never smokers (Figure 4C).

### Risk factors for COPD among current smokers, ex-smokers, and never smokers

**Figure 5:**
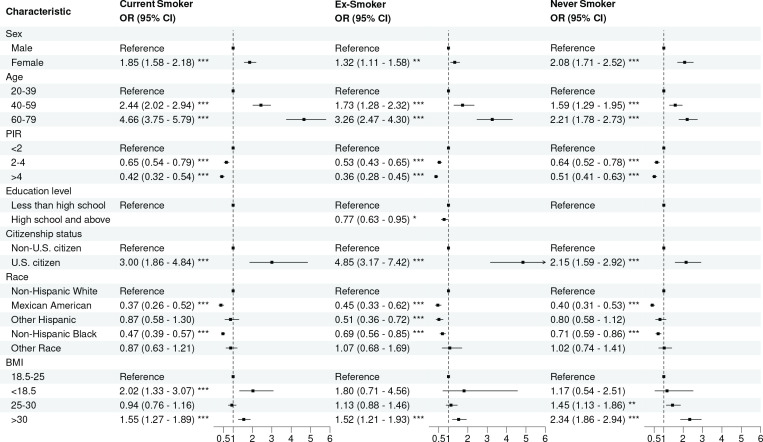
Multivariable logistic analysis of risk factors for self-reported COPD among current smokers, ex-smokers, and never smokers, aged 20–79 years, NHANES 1999–2018, United States (N=50773)

Initially, risk factors for self-reported COPD among current smokers, ex-smokers, and never smokers were identified through univariate logistic regression analysis (Supplementary file Figure 4). Subsequently, multivariate logistic regression analysis results are presented (Figure 5). Among current smokers, the following factors were identified as significant risk factors for COPD: being female (OR=1.90; 95% CI: 1.62–2.24), aged 60–79 years (OR=4.61; 95% CI: 3.71–5.73), US citizenship (OR=3.92; 95% CI: 2.48–6.22), underweight (OR=2.04; 95% CI: 1.35– 3.09), and obese (OR=1.46; 95% CI: 1.20–1.77). Among ex-smokers, the risk of COPD was found to be significantly higher in women (OR=1.32; 95% CI: 1.10–1.58), individuals aged 60–79 years (OR=3.29; 95% CI: 2.50–4.32), US citizens (OR=4.67; 95% CI: 3.06–7.12), and those classified as obese (OR=1.53; 95% CI: 1.21–1.93). Among never smokers, individuals aged 40–59 years (OR=1.59; 95% CI: 1.29–1.95) and 60–79 years (OR=2.20; 95% CI: 1.78–2.73), females (OR=2.08; 95% CI: 1.71–2.52), those with US citizenship (OR=2.15; 95% CI: 1.59– 2.92), individuals classified as overweight (OR=1.45; 95% CI: 1.13–1.86), and those classified as obese (OR=2.34; 95% CI: 1.86–2.93) exhibited a higher risk of COPD. Regardless of smoking status, whether individuals were current smokers, ex-smokers, or never smokers, higher levels of PIR were associated with a decreased risk of COPD. Additionally, among ex-smokers and non-smokers, Mexican Americans and non-Hispanic Blacks exhibit a lower risk of COPD compared to their non-Hispanic White counterparts (Figure 5).

## DISCUSSION

This research utilized data from the NHANES cross-sectional study to explore the prevalence of self-reported COPD and its associated risk factors among current smokers, ex-smokers, and non-smokers, aged 20–79 years in the US from 1999 to 2018. Additionally, cohort studies were conducted to assess all-cause mortality. The prevalence of COPD among current smokers, ex-smokers, and never smokers was 12.6%, 9.6%, and 4.1%, respectively. The observed prevalence of COPD among current smokers, ex-smokers, and never smokers may vary from findings in other studies, potentially attributable to differences in the operational definition of COPD, specifically in relation to the higher incidence of positive spirometry results. Additionally, the age demographic under investigation, which predominantly consisted of individuals aged >40 years, and variations in environmental exposures between countries, likely contributed to these differences. The risk of COPD exhibited varying degrees of susceptibility among current smokers, ex-smokers, and never smokers, highlighting the multifactorial nature of the disease. Notably, the risk of COPD was higher in women compared to men across all smoking status categories, albeit to varying extents. Females had a significantly higher risk of developing the disease compared to men, as previously documented^14,15^. A variety of physiological, environmental, and behavioral factors^16,17^ may influence this difference in risk. Older age is a significant risk factor for COPD among current smokers, ex-smokers, and never smokers, and is also associated with increased all-cause mortality in our study. COPD is characterized as a chronic condition in which a combination of genetic predisposition and environmental factors gradually contribute to structural and functional deterioration of the lungs^16^. Early life adversities, such as preterm birth, low birth weight, asthma, and infections of the lower respiratory tract during childhood, can result in irreversible impairment of lung function, potentially heightening susceptibility to developing chronic lung diseases in adulthood^17,18^. The mortality rate associated with ex-smokers with COPD was higher than that observed in current smokers with COPD. This disparity may be partially attributable to the higher prevalence of COPD among the elderly ex-smokers. The prevalence of COPD among ex-smokers and never smokers varied by race, with non-Hispanic Whites exhibiting a higher prevalence compared to other racial groups.

However, the absence of racial differences in current smoking-related COPD suggests that any potential protective advantages associated with race do not mitigate the detrimental effects of cigarette smoke exposure. This indicates that following the cessation of cigarette exposure, the variability in protective benefits among different racial groups becomes apparent. This disparity may be attributed to genetic ancestry^19^, genetic variations, and familial medical history^20,21^. Prior research has indicated that non-US citizens, or immigrants, are more likely to experience adverse behavioral, mental, and physical health outcomes that may elevate their susceptibility to illness and mortality^22^. Contrary to these findings, our research findings indicate that non-US citizens have a decreased likelihood of developing COPD, but the underlying reasons for this discrepancy remain unclear. Our study also identified a correlation between individuals with high PIR levels, indicative of high incomes, and a decreased prevalence. Previous studies have consistently demonstrated a strong association between COPD and socioeconomic status^23,24^. For example, higher wealth scores are related to a lower incidence of airway obstruction^25^, indicating that public health interventions should prioritize addressing socioeconomic disparities. Prior studies have indicated a negative correlation between education level and the prevalence of COPD among never smokers^6,26^. However, our research did not find a significant connection between education level and the prevalence of self-reported COPD among never smokers.

The prevalence and mortality rates of COPD among both current smokers and ex-smokers remain significantly high, underscoring the urgent need for enhanced preventative measures targeting these populations in the US. Conversely, the declining prevalence of COPD among never smokers over recent years suggests that the US has made notable progress in the prevention of COPD among never smokers. The prevalence and mortality rates of COPD among current smokers, ex-smokers, and never smokers remain elevated. The overall disease burden is particularly pronounced in patients with COPD who also have comorbid conditions^27^. This presents a significant challenge for healthcare providers both currently and in the future. Studies have indicated that clinical symptoms of COPD in never smokers are less pronounced compared to those in smokers^28,29^, which complicates diagnosis and increases the likelihood of the condition being overlooked. Consequently, in addition to efforts aimed at preventing COPD in current and ex-smokers, early identification of COPD in never smokers is crucial to enable timely intervention and treatment.

### Limitations

Our study has some limitations, primarily due to its cross-sectional design. This design prevents establishing a causal relationship between variables and COPD among current smokers, ex-smokers, and never smokers. Furthermore, the diagnosis of COPD was based on self-reported information and did not undergo validation through medical records. Then, social expectations, recall biases and proxy response biases can impact the accuracy of COPD prevalence estimates. Additionally, incomplete data in the NHANES, such as missing pulmonary function data (excluding the years 2007–2012), occupational exposure history, smoking intensity among smokers, and length of time since quitting among ex-smokers, also hindered the exploration of risk factors for COPD among current smokers, ex-smokers, and never smokers. Moreover, this study did not investigate the current prevalence of self-reported COPD among current smokers, ex-smokers, and never smokers from 2019 to the present, as well as acute exacerbation, due to limitations in this database. Finally, this study lacks generalizability to other countries.

## CONCLUSIONS

This study offers a comprehensive update on the epidemiology, including prevalence, mortality, and risk factors, of self-reported COPD among current smokers, ex-smokers, and never smokers within the US population aged 20–79 years from 1999 to 2018. The findings underscore that COPD continues to pose a significant public health challenge across these groups. Furthermore, the study emphasizes the critical importance of early detection and intervention in managing COPD, warranting further investigation into effective public health strategies.

## Supplementary Material



## Data Availability

The data supporting this research are available from the authors on reasonable request.

## References

[1] Blanco I, Diego I, Bueno P, Casas-Maldonado F, Miravitlles M. Geographic distribution of COPD prevalence in the world displayed by Geographic Information System maps. Eur Respir J. 2019;54(1):1900610. Published 2019 Jul 18. doi:10.1183/13993003.00610-2019 31000678

[2] World Health Organization. The top 10 causes of death. WHO; 2024. Accessed August 21, 2024. www.who.int/news-room/fact-sheets/detail/the-top-10-causes-of-death

[3] Venkatesan P. GOLD report: 2022 update. Lancet Respir Med. 2022;10(2):e20. doi:10.1016/S2213-2600(21)00561-0 34942084

[4] Agustí A, Hogg JC. Update on the pathogenesis of chronic obstructive pulmonary disease. N Engl J Med. 2019;381(13):1248-1256. doi:10.1056/NEJMra1900475 31553836

[5] Adeloye D, Song P, Zhu Y, et al. Global, regional, and national prevalence of, and risk factors for, chronic obstructive pulmonary disease (COPD) in 2019: a systematic review and modelling analysis. Lancet Respir Med. 2022;10(5):447-458. doi:10.1016/S2213-2600(21)00511-7 35279265 PMC9050565

[6] Yang IA, Jenkins CR, Salvi SS. Chronic obstructive pulmonary disease in never-smokers: risk factors, pathogenesis, and implications for prevention and treatment. Lancet Respir Med. 2022;10(5):497-511. doi:10.1016/S2213-2600(21)00506-3 35427530

[7] Celli B, Fabbri L, Criner G, et al. Definition and nomenclature of chronic obstructive pulmonary disease: time for its revision. Am J Respir Crit Care Med. 2022;206(11):1317-1325. doi:10.1164/rccm.202204-0671PP 35914087 PMC9746870

[8] Terzikhan N, Verhamme KM, Hofman A, Stricker BH, Brusselle GG, Lahousse L. Prevalence and incidence of COPD in smokers and non-smokers: the Rotterdam Study. Eur J Epidemiol. 2016;31(8):785-792. doi:10.1007/s10654-016-0132-z 26946425 PMC5005388

[9] Lee SH, Hwang ED, Lim JE, et al. The risk factors and characteristics of COPD among nonsmokers in Korea: an analysis of KNHANES IV and V. Lung. 2016;194(3):353-361. doi:10.1007/s00408-016-9871-6 27038474

[10] Ivey MA, Smith SM, Benke G, et al. COPD in never-smokers: BOLD Australia Study. Int J Chron Obstruct Pulmon Dis. 2024;19:161-174. doi:10.2147/COPD.S439307 38249822 PMC10800088

[11] National Center for Health Statistics, Centers for Disease Control and Prevention. NCHS Ethics Review Board (ERB) Approval. CDC. Accessed August 21, 2024. https://www.cdc.gov/nchs/nhanes/irba98.htm

[12] National Center for Health Statistics, Centers for Disease Control and Prevention. About the National Health and Nutrition Examination Survey. CDC. Accessed August 21, 2024. https://www.cdc.gov/nchs/nhanes/about_nhanes.htm

[13] Centers for Disease Control and Prevention. 2015-2016 Data Documentation, Codebook, and Frequencies. CDC; 2017. Accessed August 21, 2024. https://wwwn.cdc.gov/Nchs/Nhanes/2015-2016/DEMO_I.htm

[14] Lamprecht B, McBurnie MA, Vollmer WM, et al. COPD in never smokers: results from the population-based burden of obstructive lung disease study. Chest. 2011;139(4):752-763. doi:10.1378/chest.10-1253 20884729 PMC3168866

[15] Montserrat-Capdevila J, Godoy P, Marsal JR, et al. Prevalencia y características de la enfermedad pulmonar obstructiva crónica en no fumadores. Aten Primaria. 2019;51(10):602-609. doi:10.1016/j.aprim.2017.10.012 30454958 PMC6930941

[16] Agustí A, Melén E, DeMeo DL, Breyer-Kohansal R, Faner R. Pathogenesis of chronic obstructive pulmonary disease: understanding the contributions of gene-environment interactions across the lifespan. Lancet Respir Med. 2022;10(5):512-524. doi:10.1016/S2213-2600(21)00555-5 35427533 PMC11428195

[17] Bush A. Impact of early life exposures on respiratory disease. Paediatr Respir Rev. 2021;40:24-32. doi:10.1016/j.prrv.2021.05.006 34144911

[18] Savran O, Ulrik CS. Early life insults as determinants of chronic obstructive pulmonary disease in adult life. Int J Chron Obstruct Pulmon Dis. 2018;13:683-693. doi:10.2147/COPD.S153555 29520136 PMC5834168

[19] Bruse S, Sood A, Petersen H, et al. New Mexican Hispanic smokers have lower odds of chronic obstructive pulmonary disease and less decline in lung function than non-Hispanic whites. Am J Respir Crit Care Med. 2011;184(11):1254-1260. doi:10.1164/rccm.201103-0568OC 21908412 PMC3262041

[20] Moll M, Lutz SM, Ghosh AJ, et al. Relative contributions of family history and a polygenic risk score on COPD and related outcomes: COPDGene and ECLIPSE studies. BMJ Open Respir Res. 2020;7(1):e000755. doi:10.1136/bmjresp-2020-000755 PMC7689586 33239407

[21] Sikjær MG, Klitgaard A, Hilberg O, Løkke A. Parental COPD as a risk factor for the development of COPD and disease severity in offspring: a systematic scoping review. Int J Chron Obstruct Pulmon Dis. 2022;17:1323-1338. doi:10.2147/COPD.S364899 35706707 PMC9188979

[22] Guillot-Wright S, Cherryhomes E, Wang L, Overcash M. Systems and subversion: a review of structural violence and im/migrant health. Curr Opin Psychol. 2022;47:101431. doi:10.1016/j.copsyc.2022.101431 36001924

[23] Raju S, Keet CA, Paulin LM, et al. Rural residence and poverty are independent risk factors for chronic obstructive pulmonary disease in the United States. Am J Respir Crit Care Med. 2019;199(8):961-969. doi:10.1164/rccm.201807-1374OC 30384774 PMC6467317

[24] Patel JH, Amaral AFS, Minelli C, et al. Chronic airflow obstruction attributable to poverty in the multinational Burden of Obstructive Lung Disease (BOLD) study. Thorax. 2023;78(9):942-945. doi:10.1136/thorax-2022-218668 37423762 PMC10954321

[25] Townend J, Minelli C, Mortimer K, et al. The association between chronic airflow obstruction and poverty in 12 sites of the multinational BOLD study. Eur Respir J. 2017;49(6):1601880. doi:10.1183/13993003.01880-2016 28572124

[26] Oh H, Lee YE. Prevalence and risk factors of chronic obstructive pulmonary disease among nonsmokers: fifth Korea National Health and Nutrition Examination Survey (2010-2012). Osong Public Health Res Perspect. 2016;7(6):385-393. doi:10.1016/j.phrp.2016.11.006 28053845 PMC5194221

[27] Mariniello DF, D’Agnano V, Cennamo D, et al. Comorbidities in COPD: current and future treatment challenges. J Clin Med. 2024;13(3):743. doi:10.3390/jcm13030743 38337438 PMC10856710

[28] Tan WC, Sin DD, Bourbeau J, et al. Characteristics of COPD in never-smokers and ever-smokers in the general population: results from the CanCOLD study. Thorax. 2015;70(9):822-829. doi:10.1136/thoraxjnl-2015-206938 26048404

[29] Yang IA, Jenkins CR, Salvi SS. Chronic obstructive pulmonary disease in never-smokers: risk factors, pathogenesis, and implications for prevention and treatment. Lancet Respir Med. 2022;10(5):497-511. doi:10.1016/S2213-2600(21)00506-3 35427530

