# Supplementary material for: Prevalence, mortality and risk factors for self-reported COPD among smokers and never smokers, NHANES 1999-2018
Source: Tob Induc Dis. 2024 Sep 18;22:10.18332/tid/192745. doi: 10.18332/tid/192745 (PMC11409450; doi:10.18332/tid/192745)
Supplement: Supplementary file 1 [file TID-22-161-s1.pdf]

Supplement of method

1. logistic regression analysis:

In this study, we constructed separate logistic regression models for the never smokers, ex-smokers, and current smokers.

(1) logistic regression models for the never smoker group：

Based on the outcomes of univariate logistic regression analysis for never smokers group, variables exhibiting significant associations with the prevalence of COPD were identified. In this investigation, the prevalence of COPD was designated as the dependent variable, while RIAGENDR (Sex), RIDAGEYR (Age), RIDRETH1 (Race/ethnicity), DMDCITZN (Citizenship status), INDFMPIR (PIR), BMXBMI (BMI), and DMDEDUC2 (Education level) were designated as independent variables. The sequential construction of multiple logistic regression models was carried out using the svyglm function of the survey package.

Table 1. Multiple logistic regression models for the never smokers group

model	independent variable	model formula
**model1**	RIAGENDR, RIDAGEYR, RIDRETH1	logit(P(COPD)) = β0 + β1 * RIAGENDR + β2 * RIDAGEYR + β3 * RIDRETH1
**model2**	RIAGENDR, RIDAGEYR, RIDRETH1, DMDCITZN	logit(P(COPD)) = β0 + β1 * RIAGENDR + β2 * RIDAGEYR + β3 * RIDRETH1 + β4 * DMDCITZN
**model3**	RIAGENDR, RIDAGEYR, RIDRETH1, DMDCITZN, INDFMPIR, BMXBMI	logit(P(COPD)) = β0 + β1 * RIAGENDR + β2 * RIDAGEYR + β3 * RIDRETH1 + β4 * DMDCITZN + β5 * INDFMPIR + β6 * BMXBMI
**model4**	RIAGENDR, RIDAGEYR, RIDRETH1, DMDCITZN, INDFMPIR, BMXBMI, DMDEDUC2	logit(P(COPD)) = β0 + β1 * RIAGENDR + β2 * RIDAGEYR + β3 * RIDRETH1 + β4 * DMDCITZN + β5 * INDFMPIR + β6 * BMXBMI + β7 * DMDEDUC2

Abbreviations: Logit, logit function

We perform likelihood ratio tests between model pairs: model1 vs model2, model2 vs model3, and model3 vs model4. The objective of these likelihood ratio tests is to compare the goodness-of-fit between nested logistic regression models. Specifically, these tests aim to ascertain whether the inclusion of additional parameters in the more complex model results in a statistically significant improvement in model fit. By evaluating the difference in the log-likelihood values of the models and conducting hypothesis tests, we can determine the significance of the enhancements. This methodological approach allows us to identify the variables that contribute substantively to the model, ensuring that the model achieves an optimal balance between explanatory power and parsimony, thereby mitigating the risk of overfitting.

The likelihood ratio test comparing model1 to model2 (2logLR=36.53794, p= 0.000000001654<0.05) indicates statistically model2 provides a better fit to the data than model1, which is a reduced model of model2. And it indicates after adding the DMDCITZN variable, the fitting effect of the logistic regression model for COPD was significantly improved compared to model1. The likelihood ratio test comparing model 2 to model 3 (2logLR = 36.53794 ,p= 0.000000001654 <0.05) also indicates a statistically significant difference between the two models. However, the likelihood ratio test showed that model4 is not significantly different from model3 in fitting the data for COPD (2logLR = 0.2049948， p= 0.64556), so it can

be shown that model3 is sufficient for fitting COPD. In further study, A model test was conducted for model3, and the parameter test results are displayed in Table 2.

Table 2. Test results of model3 for never smoker group

	STIMATE	TD. ERROR	VALUE	R(>|T|)	
INTERCEPT)	4.693961129	.210579478	22.29068656	.0541e-109	**
IAGENDR2	.730363881	.099077709	.371626686	.74102E-13	**
IDAGEYR2	.463769147	.104940876	.419337504	.94447E-06	**
IDAGEYR3	.791782501	.108961018	.266658437	.79934E-13	**
IDRETH11	0.909787263	.136624081	6.65905494	.81646E-11	**
IDRETH12	0.219595547	.168240363	1.30524889	.19182083	
IDRETH14	0.34357011	.097124537	3.537418252	.000404834	**
IDRETH15	.020278493	.164566456	.123223731	.901930983	
MDCITZN1	.765809703	.155384825	.928471623	.34366E-07	**
NDFMPIR2	0.452621981	.104163314	4.345310864	.39659E-05	**
NDFMPIR3	0.672490264	.108972835	6.171173413	.88877E-10	**
MXBMI1	.154079781	.391469946	.393592873	.693885198	
MXBMI3	.371504909	.126989047	.925487807	.00344244	*
MXBMI4	.849404741	.116129349	.314298655	.66977E-13	**

when p value is smaller than 0.05 for parameter estimate of some level of the categorical variable, we considered the level to have a statistically significant effect on the prevalence of COPD compared with the reference level. As can be seen from the figure, all independent variables have some level that have a significant impact on the prevalence of COPD compared with their reference level, which means that all of the variable in model3 is statistically significant. In addition, we tested whether there is multicollinearity in the model3, and the calculated VIF values are shown in Table 3 below.

Table 3. VIF values of modle3 for never smoker group

	GVIF	*DF*	*GVIF^(1/(2*Df))*
IAGENDR	1.083486512		1.040906582
IDAGEYR	1.153494676		1.036343908
IDRETH1	1.511086659		1.052958298
MDCITZN	1.2049655		1.097709205
NDFMPIR	1.220450076		1.051066041
MXBMI	1.227520162		1.034756363

Abbreviations: GVIF, Generalized Variance Inflation Factor; Df, Degrees of Freedom

According to the data presented in Table 2, none of the values for the generalized variance inflation factor and the modified generalized variance inflation factor exceed 2, suggesting there is no multicollinearity in model3.

In conclusion, when the variables in model3 vary at different levels, they all exhibit significant differences in their impact on the prevalence of COPD compared to the reference level. Moreover, there is no evidence of multicollinearity among the independent variables in model3, and as a reduced model of model4, model3 has provided an adequate fit to the data. Therefore, we select model3 as the final logistic regression model for estimating the prevalence of COPD.

(2) logistic regression models for the ex-smoker group：

Similar to the modeling process for logistic regression models for never smokers, we established four nested models (Model 1 to Model 4) for the ex-smokers group as outlined in Table 1. We conducted pairwise comparisons of the model fits using the likelihood ratio test. The results are presented in Table 4.

Table 4. Likelihood ratio test for logistic regression models of ex-smoker group

	**2LOGLR**	**P_VALUE**
**MODEL1 VS MODEL2**	44.52828	2.78e-11
**MODEL2 VS MODEL3**	115.3608	<2.22e-16
**MODEL3 VS MODEL4**	5.573745	0.01904

2logLR: twice the log-likelihood ratio

Table 4 shows that Model 4 significantly outperforms Models 1, 2, and 3, as the likelihood ratio tests for all four comparisons rejected the null hypothesis at the 0.05 significance level. This indicates that the more complex model is significantly better than the simpler models in each of

the four tests, with Model 4 being the most complex of the four. Furthermore, a model test was conducted for Model 4 in the ex-smoker group, and the results are presented in Table 5.

Table 5. Model test for model4 of ex-smoker group

	STIMATE	TD. ERROR	VALUE		
**INTERCEPT)**	4.0332	.27241	14.806	2e-16	**
**IAGENDR2**	.28066	.0894	.139	.001698	*
**IDAGEYR2**	.54639	.15147	.607	.000311	**
**IDAGEYR3**	.18178	.14114	.373	2e-16	**
**IDRETH11**	0.79557	.16242	4.898	.82E-07	**
**IDRETH12**	0.67699	.17755	3.813	.000138	**
**IDRETH14**	0.37168	.10524	3.532	.000415	**
**IDRETH15**	.06688	.23364	.286	.774692	
**MDCITZN1**	.57797	.21714	.267	.94E-13	**
**NDFMPIR2**	0.63702	.10607	6.006	.97E-09	**
**NDFMPIR3**	1.02886	.11865	8.671	2e-16	**
**MXBMI1**	.59039	.47318	.248	.212171	
**MXBMI3**	.12279	.12892	.952	.340868	
**MXBMI4**	.42145	.11924	.535	.00041	**
**MDEDUC22**	0.25493	.10653	2.393	.016728	

Table 5 shows that, Similar to model3 for never smoker group, all independent variables of model4 for ex-smoker group have at least one level with a significant impact on the prevalence of COPD relative to their reference levels. And the VIF values of molde4 in Table 6 below suggests there is no multicollinearity in model3 for . As a result, model4 is determined to be the final logistic model for ex-smoker group ex-smoker group.

Table 6. VIF values of modle4 for never smoker group

	VIF	F	GVIF^(1/(2*DF))
R<br>IAGENDR	.034149		.016931
R<br>IDAGEYR	.163361		.038553
R<br>IDRETH1	.377342		.040831
D<br>MDCITZN	.156787		.07554
I<br>NDFMPIR	.238093		.054844
B<br>MXBMI	.158441		.024815
D<br>MDEDUC2	.204509		.097501

(3) logistic regression models for the current smokers：

Similarly, we developed four distinct logistic regression models for the current smoker group, as outlined in Table 1. In line with the approach used for selecting the final model for the never smoker group, we applied the likelihood ratio test, the Score Test, and examined the Variance Inflation Factor (VIF) to finalize the model for current smokers. Based on these assessments, Model 3 was selected as the final model. The results of the likelihood ratio test, the Score Test, and VIF for Model 3 are provided in Tables 7 through 9.

Tables 7 .model test for model3 of current smoker group

	2LOGLR	P_VALUE
MODEL1 VS MODEL	28.28589	1.1482e-07
MODEL2 VS MODEL3	99.94097	<2.22e-16
MODEL3 VS MODEL4	3.449921	0.065173

2logLR: twice the log-likelihood ratio

Tables 8. Model test for model3 of current smoker group

	stimate	td. Error	value	r(>|t|)
Intercept)	3.72041	.26075	14.268	2e-16 ***
IAGENDR2	.61732	.08293	.443	.06e-13 ***

IDAGEYR2	.89036	.09526	.347	2e-16 ***
IDAGEYR3	.53858	.11081	3.884	2e-16 ***
MDCITZN1	.09893	.24437	.497	.97e-06 ***
NDFMPIR2	0.42837	.09866	4.342	.43e-05 ***
NDFMPIR3	0.87384	.12897	6.776	.31e-11 ***
MXBMI1	.70471	.21308	.307	.000946 ***
MXBMI3	0.06717	.1087	0.618	.536642
MXBMI4	.438	.10004	.378	.21e-05 ***
IDRETH11	0.9958	.17075	5.832	.66e-09 ***
IDRETH12	0.14319	.20605	0.695	.48713
IDRETH14	0.74632	.09433	7.912	.81e-15 ***
IDRETH15	0.13454	.1646	0.817	.413737

Table 9. VIF values of modle3 for current smoker group

	GVIF
RIAGENDR	1.045458
RIDAGEYR	1.075506
DMDCITZN	1.120809
INDFMPIR	1.088392
BMXBMI	1.087963
RIDRETH1	1.230487

Prevalence of COPD in Current-Smokers

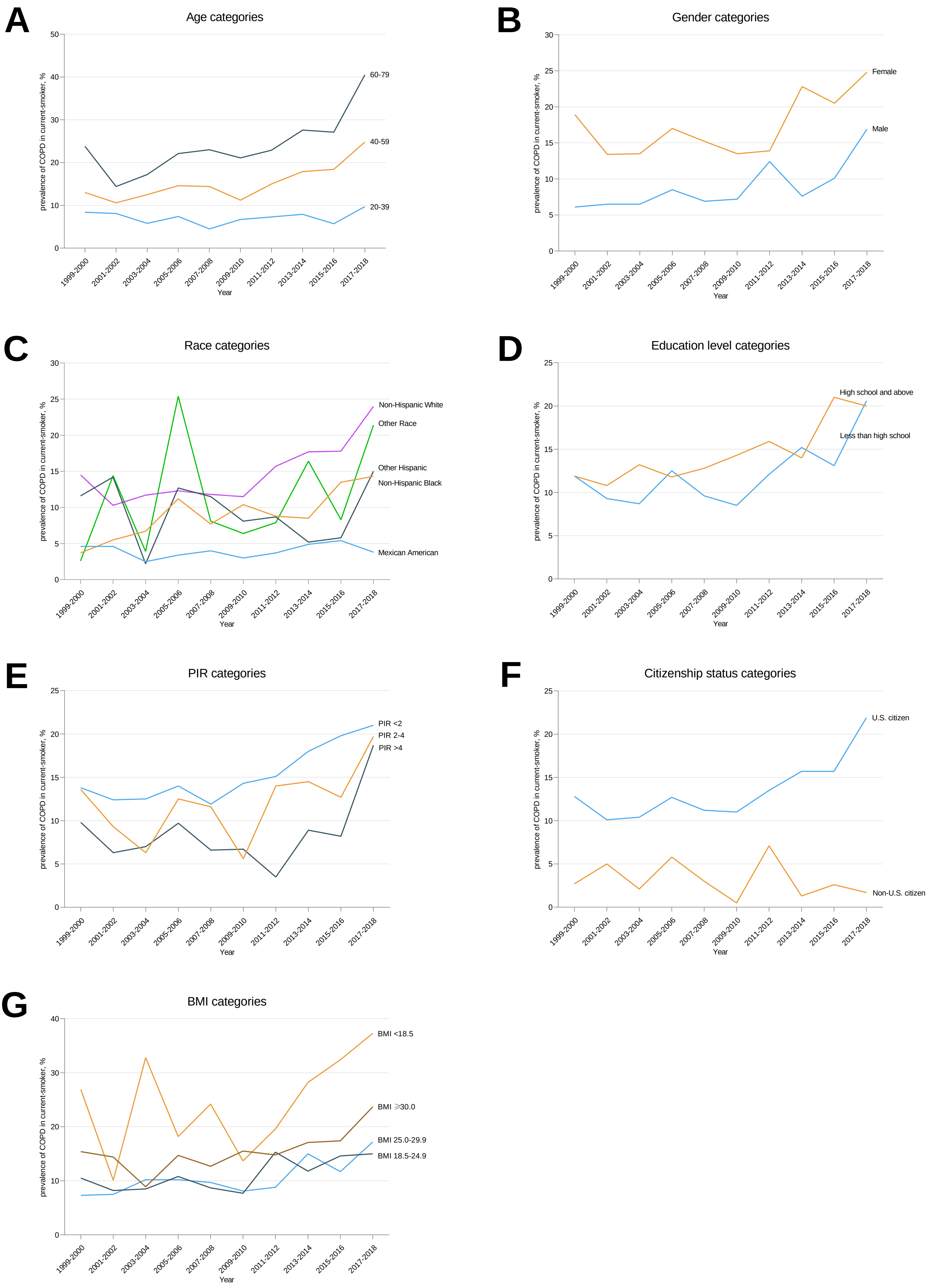

Prevalence of COPD in Ex-Smokers

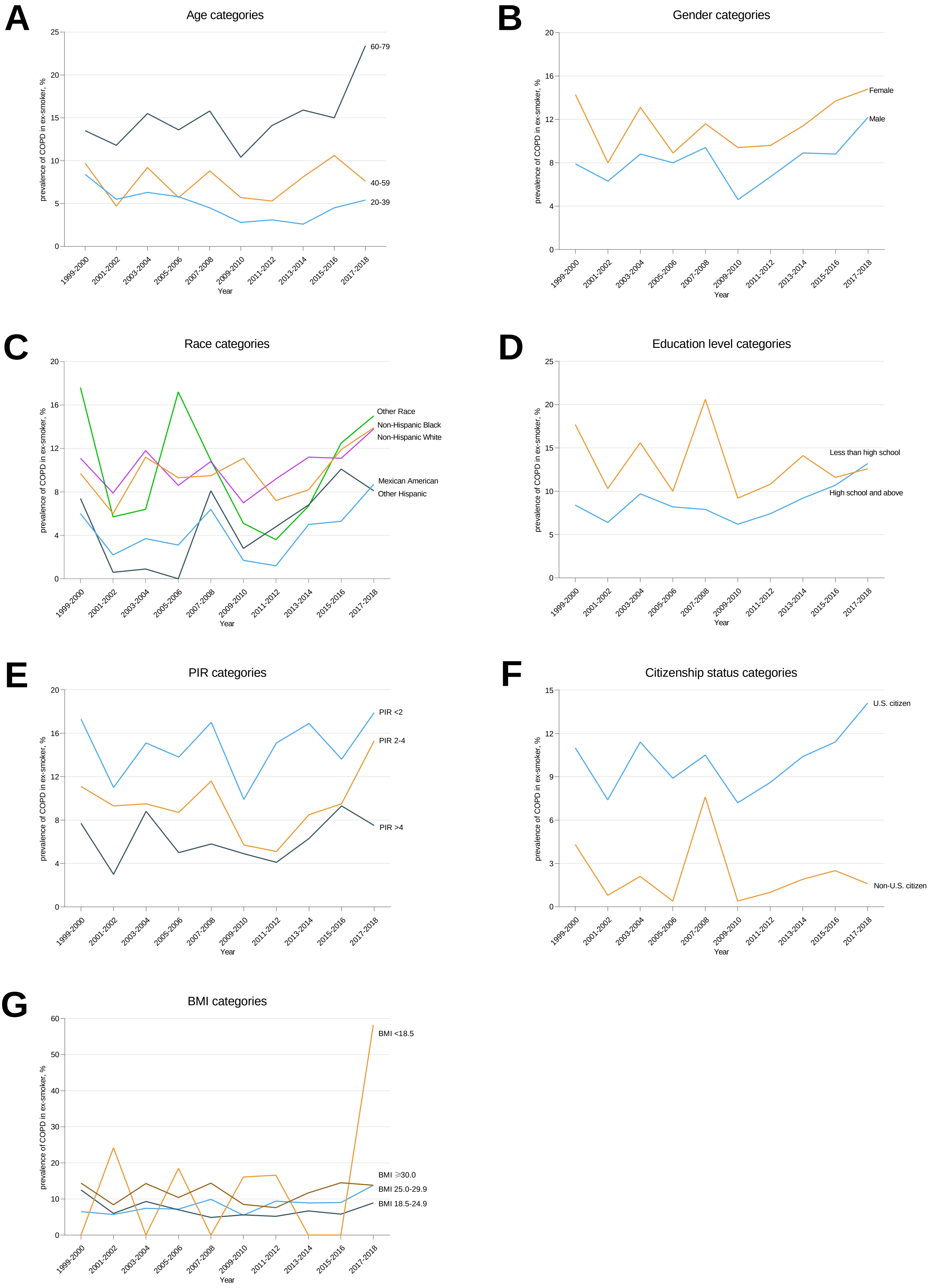

Prevalence of COPD in Never-Smokers

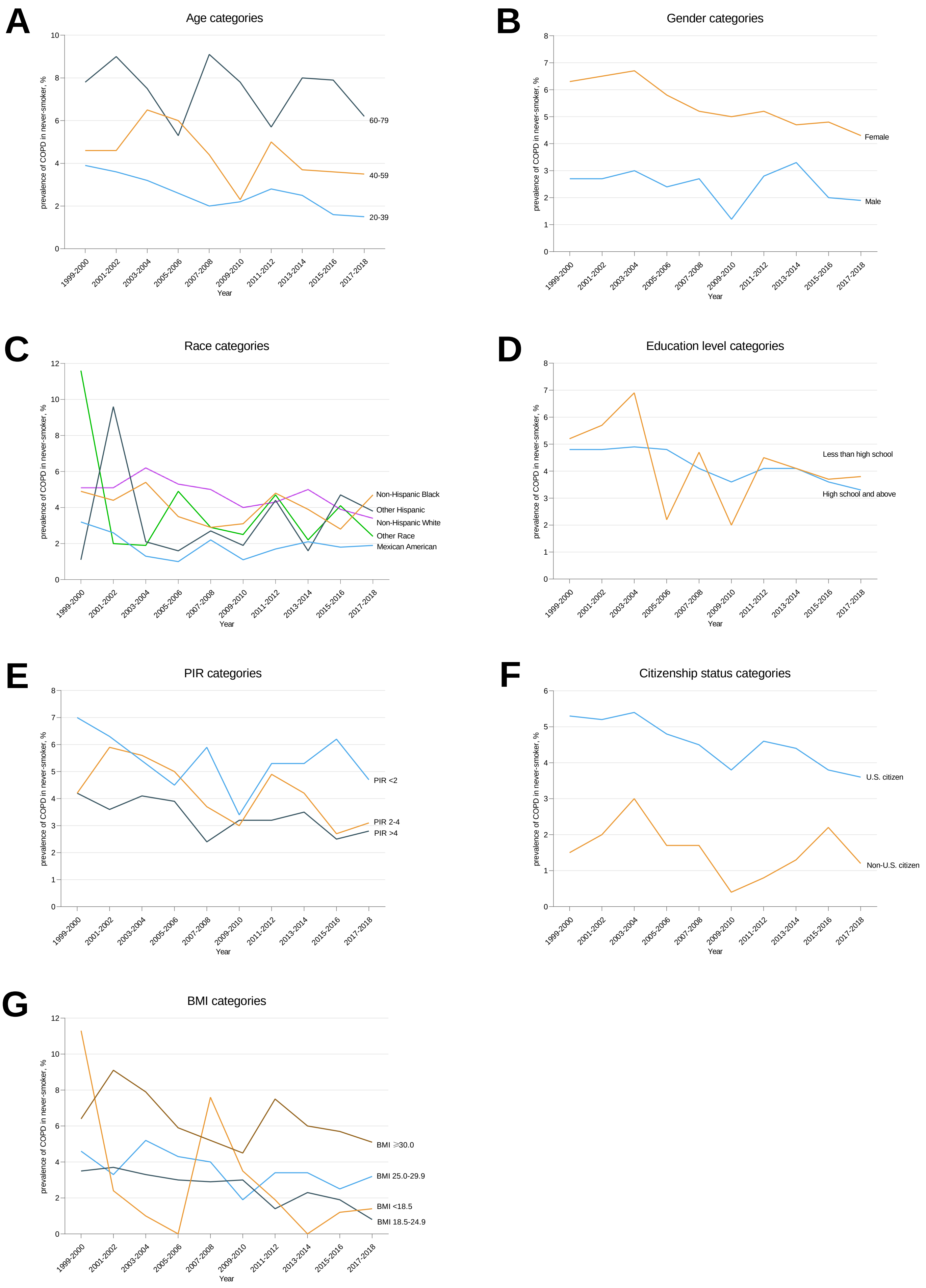

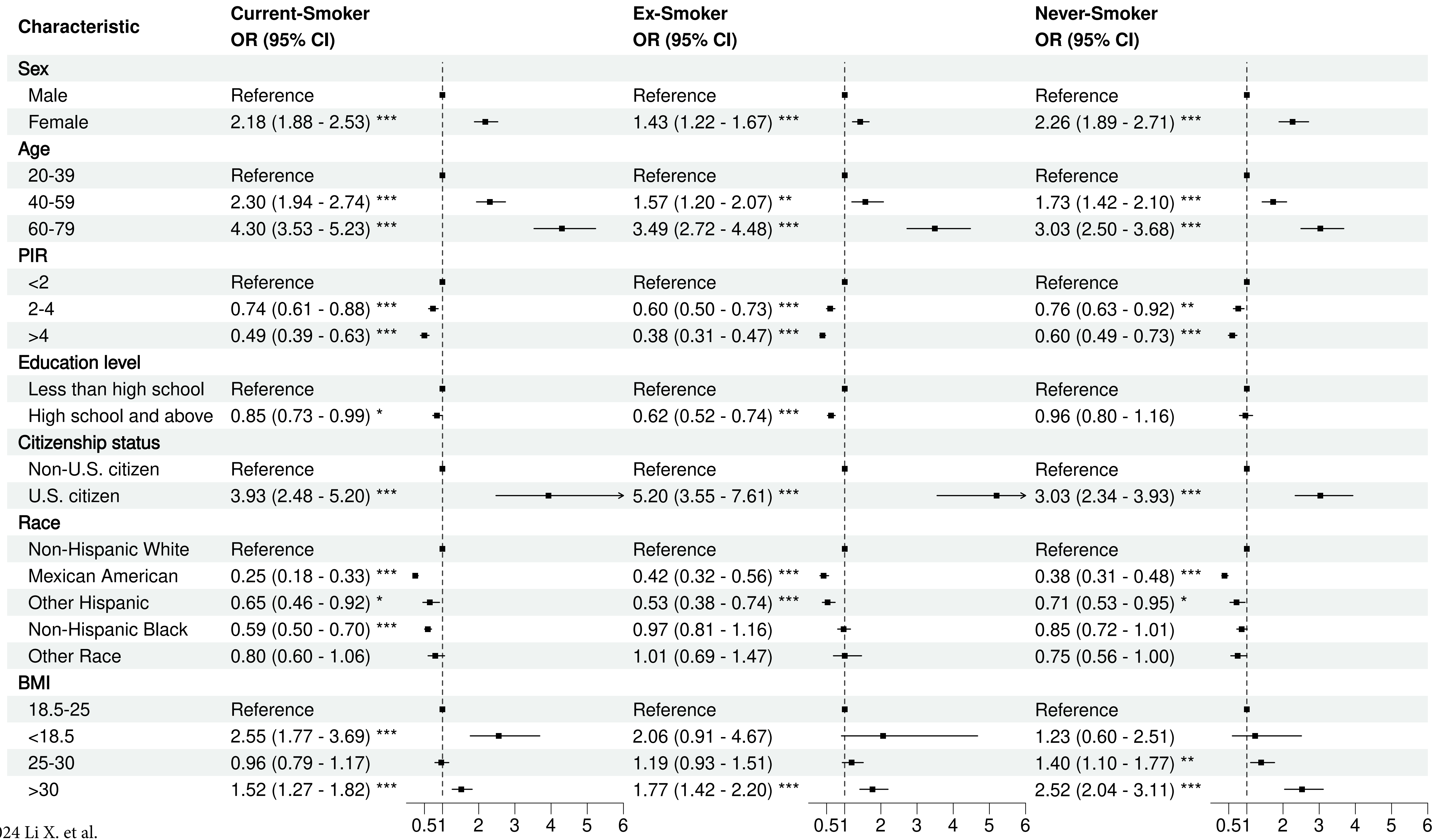

Characteristic	Current-Smoker OR (95% CI)	Ex-Smoker OR (95% CI)	Never-Smoker OR (95% CI)
**Sex**			
Male	Reference	Reference	Reference
Female	2.18 (1.88 - 2.53) ***	1.43 (1.22 - 1.67) ***	2.26 (1.89 - 2.71) ***
**Age**			
20-39	Reference	Reference	Reference
40-59	2.30 (1.94 - 2.74) ***	1.57 (1.20 - 2.07) **	1.73 (1.42 - 2.10) ***
60-79	4.30 (3.53 - 5.23) ***	3.49 (2.72 - 4.48) ***	3.03 (2.50 - 3.68) ***
**PIR**			
<2	Reference	Reference	Reference
2-4	0.74 (0.61 - 0.88) ***	0.60 (0.50 - 0.73) ***	0.76 (0.63 - 0.92) **
>4	0.49 (0.39 - 0.63) ***	0.38 (0.31 - 0.47) ***	0.60 (0.49 - 0.73) ***
**Education level**			
Less than high school	Reference	Reference	Reference
High school and above	0.85 (0.73 - 0.99) *	0.62 (0.52 - 0.74) ***	0.96 (0.80 - 1.16)
**Citizenship status**			
Non-U.S. citizen	Reference	Reference	Reference
U.S. citizen	3.93 (2.48 - 5.20) ***	5.20 (3.55 - 7.61) ***	3.03 (2.34 - 3.93) ***
**Race**			
Non-Hispanic White	Reference	Reference	Reference
Mexican American	0.25 (0.18 - 0.33) ***	0.42 (0.32 - 0.56) ***	0.38 (0.31 - 0.48) ***
Other Hispanic	0.65 (0.46 - 0.92) *	0.53 (0.38 - 0.74) ***	0.71 (0.53 - 0.95) *
Non-Hispanic Black	0.59 (0.50 - 0.70) ***	0.97 (0.81 - 1.16)	0.85 (0.72 - 1.01)
Other Race	0.80 (0.60 - 1.06)	1.01 (0.69 - 1.47)	0.75 (0.56 - 1.00)
**BMI**			
18.5-25	Reference	Reference	Reference
<18.5	2.55 (1.77 - 3.69) ***	2.06 (0.91 - 4.67)	1.23 (0.60 - 2.51)
25-30	0.96 (0.79 - 1.17)	1.19 (0.93 - 1.51)	1.40 (1.10 - 1.77) **
>30	1.52 (1.27 - 1.82) ***	1.77 (1.42 - 2.20) ***	2.52 (2.04 - 3.11) ***

© 2024 Li X. et al.
